# The Microscopical Aspect of Certain Lesions Induced by Dental Caries

**Published:** 1897-06

**Authors:** A. Hopewell Smith

**Affiliations:** L.R.C.P. (Lond.), M.R.C.S., L.D.S. (Eng.)


### Abstracts and Translations.

### THE MICROSCOPICAL ASPECT OF CERTAIN LESIONS INDUCED BY DENTAL CARIES.

BY A. HOPEWELL SMITH, L.R.C.P. (LOND.), M.R.C.S., L.D.S. (ENG.).

It would save a great confusion of ideas if three kinds of dentinal deposition were generally recognized: (1) Calcareous degeneration of the pulp, a constant accompaniment of caries, but also found occasionally in sound teeth as the result of vascular changes due to idiopathic or constitutional causes; (2) secondary dentine, occurring not only as a pathological process in cases of attrition, abrasion, or fracture, but physiologically, as the result of senile changes in both permanent and long-retained deciduous teeth; and (3) adventitious dentine, the product of caries.

Salter's[1] patient and remarkable investigations in this particular portion of dental pathology led him to classify all forms of dentine deposition as secondary dentine, and in a sense this was perfectly correct.

But the term seems to require a more definite meaning; for he describes under this one heading three forms,—viz., dentine of repair, dentine excrescence, and osteo-dentine or intrinsic calcification of the pulp.

The adjoining scheme is intended to show the chief results of pathological changes taking place in the dentine and the pulp of a tooth.

---

[1] Dental Pathology and Surgery, chap. xi. and xii., 1874.

| | |
|---|---|
| I. *Calcareous degeneration of pulp*, through changes in pulp alone. | (*a*) Common. Due to vascular changes in pulp, in association with caries.<br>(*β*) Rare. Due to idiopathic or constitutional changes,—*e.g.*, gout. |
| II. *Secondary dentine in pulp-cavity*, through changes in the primary dentine and pulp. | (*a*) Physiological. Due to senile conditions of both permanent and long-retained deciduous teeth.<br>(*β*) Pathological. Due to attrition, abrasion, or fracture. |
| III. *Adventitious dentine in pulp-cavity*, through changes in the primary dentine and pulp. | Due to caries. |

Peripheral carious stimulation of the dentine is accompanied by destructive as well as constructive metamorphoses; tissue waste and tissue repair go on side by side. At first the soft parts alone suffer; the dentinal fibrils and their enclosing tubules, in parts of their courses, are affected and soon become disorganized, the blood-vessels and tissues of the pulp undergo hyperæmic and other changes. Thus a superficial carious patch beneath the enamel is associated with marked cellular activity on the part of the pulp; while there is a loss of substance externally, there is a gain internally. This is exemplified in the formation under certain circumstances of "dentine of repair." In other words, caries, even in its early stages, usually leads to a deposit of new adventitious dentine on the surface of the pulp.

But later on bacterial agencies multiply and accumulate, advancement renders them still more potent, and development means destruction. For now not only do the dentinal tubules and matrix also become involved in the general dissolution, but any adventitious tissues that may have been developed rapidly break down, and soon the work of demolition is complete. A study of these phenomena possesses several points of profound interest.

### (A) *Conditions associated with Superficial Caries.*

Viewed from a clinical aspect, it may be said that the commencement of caries is marked, as a rule, by one of two distinct types of lesions: (1) the not uncommon clean-cut cavity which by its general appearance suggests erosion; and (2) the usual cavity of decay. The former is distinguished by its position on the cervical portion of the labial aspect of the anterior teeth, and by its intense hyperæsthesia on receiving interrupted tactile impressions; the latter is recognized by its inability to transmit slight functional impulses to the pulp. Microscopically the difference between these two classes is well defined, the first named particularly presenting marked deviations from the usual type of decay.

(1) Here the subenamel region of the dentine contains not only the usual "granular layer," but also areas occupied by large interglobular spaces, which are distributed with more or less regularity throughout its substance. Micro-organisms are present in enormous numbers at the margin of the cavity, and fill the tubules for varying distances. Opposite the breach of surface a corresponding deposit of adventitious dentine with enlarged irregular tubules is observed. The layer of tissue "on the borderland of calcification" is increased in thickness and exhibits a greater quantity of calcoglobular masses than normally. They are, however, very small. In the pulp, slight hyperæmia and cell-proliferation have certainly occurred in this locality and its neighborhood; and the peripheral cells, which present many of the appearances of the so-called odontoblasts, are multiplied greatly. Beyond, the tissues may be considered to be normal, with the exception, perhaps, of the smaller blood-vessels, whose lamina are more or less increased in size. Rounded cylindrical deposits of new dentine constantly exist in the central portions of the pulp, and point to a degenerative process. The changes from the normal to the pathological areas are very gradual, no sharp line of demarcation cutting them off from the other parts of the soft tissues.

Referring to the statement just enunciated that "the so-called odontoblasts are multiplied greatly," it must not be inferred that these cells are merely increased in point of numbers. They are profoundly modified, inasmuch as they now possess certain new characteristics. Their nuclei have become elongated and flattened, and are rendered very prominent when any of the nuclear stains have been used, and perhaps they are more granular than usual. The cell walls are indistinguishable, chiefly from the fact that each odontoblast is compressed laterally by its neighbors. In some instances they are gathered into sheaves. Some observers might describe the appearances as being due to an indirect splitting-up of the cells, and it is not difficult to conceive an odontoblast, when once fully formed, undergoing the process of karyokinesis. In sections prepared by Weil's method (and for this the writer's best thanks are due to Mr. W. H. Must), macroscopically the tubules in the primary dentine below the breach of surface are unaffected by stains, and clearly differentiated from other tubules, and a band of altered pulp-tissue extends right across that organ.

Thus at the very outset we meet with two most remarkable conditions, and our attention is immediately arrested by their presence. These are the multiplication of the numbers of interglobular spaces, and also of the so-called odontoblast cells,—phenomena which are entirely absent from all ordinary conditions.

There would, therefore, seem to be some connection between the subjective symptoms of pain and these fresh developments,—or at all events one of those fresh developments,—and this leads one to the conclusion that the degree of the sensitiveness of these cavities is dependent chiefly on the increase or diminution in the numbers of the interglobular spaces.

Bödecker,[1] in speaking of dental irritation in the case of ordinary caries, attributes the sensations of pain to "alternate contractions and expansions of living matter" in dentine and enamel, "conveyed from the periphery to the centre of the tooth, these intense contractions being induced by highly irritating agencies."

But it must be remembered that it is mechanical stimulation alone of the floor of these particular cavities that gives rise to pain; and we must infer, with greater accuracy in the light of our knowledge of the physiological stimulation of nerves and protoplasm generally, that the pathic disturbances are due here to direct impulses, which pass by means of the dentinal fibrils from the protoplasmic contents of the interglobular spaces to the ultimate ramifications of the sensory pulp nerves.

The occurrence of additions to the numbers of the long odontoblast pulp-cells does not admit of quite so easy an explanation, and on this question the opinions of those who have worked at this branch of the subject would be welcomed. The American author[2] just quoted has indirectly noticed, although he has not figured this phenomenon. He says, "The first change in the affected pulp-tissue is its reduction to an embryonal or protoplasmic state,"—a statement which is certainly not verified on examination of my own microscopic preparations. Further, he writes, "Should the lymph-tissue be reduced to its embryonal conditions as above indicated, the protoplasm present before transformation into basis-substance reappears, and *may break up into odontoblasts* or into *osteoblasts.* In the former case, the result of irritation of the pulp-tissue will be

---

[1] Anatomy and Pathology of the Teeth, 1894, p. 288. [2] Op. cit.

*dentine*, in the latter *bone*." The method of thus interpreting the genesis of these lime-bearing cells is crude and illogical.

True bony formations are most rarely found in the tooth-pulp; such a case, however, has been recorded by Messrs. J. F. Colyer and Ackery,[1] but calcareous deposits are exceedingly common.

Compact osseous tissue consists of Haversian canals, concentric, and intermediary lamellæ, lacunæ, and canaliculi, with blood-vessels, osteoblasts, connective tissue, branched bone-corpuscles, and minute lymphatic systems. And if these component parts are non-existent, it is a mistake to pronounce the new formation bone.

The local increase in the numbers of the odontoblasts may show that, in certain situations, there is a greater need for the higher and more sustained exercise of their functions, these functions, in my opinion, consisting wholly of shielding the delicate pulp from incoming dangers; not by the production of dentine matrix, but by physiologically creating a larger or more concentrated area of trophic influence or control—if one may so speak—on the surface of that organ, whereby its vitality may be retained until the latest possible moment; or it may be that the odontoblasts have merely undergone cell-subdivision. This is probably the correct view to hold.

(2) Turning, in the second place, to cases in which the dentine is well-developed and free from irregularities, the subjective pain symptoms do not, as a rule, appear until there is almost penetration into the pulp-chamber, no matter how rapidly the carious encroachments may take place. But the pulp exhibits similar microscopical characteristics to those already detailed, the most obvious being cell-proliferation and odontoblast multiplication with isolated cylindrical dentine formations in the neighborhood of the vessels. Regional hyperæmia[2] is often present,—that is, the capillaries and blood-vessels are rather larger and have thinner walls than normal.

Pain here is undoubtedly due to lateral pressure on the nerve bundles, the chief factors being the hyperæmia of the blood-vessels, the cellular infiltration of the tissues, and the presence of new dentine nodules.[3] If inflammation supervenes, pain is greatly intensi-

---

[1] Transactions of the Odontological Society of Great Britain, vol. xxv., No. 3.

[2] "Regional hyperæmia" is a term used in this connection to denote a localized partial hyperæmic condition of the blood-vessels. Thus one may speak of "coronal" or "cornual," "cervical," "radicular hyperæmia," according to its situation.

[3] See Sewill, Dental Surgery, 1890, p. 234.

fied by pressure on the pulp-tissues of the serous exudation from the vessels. It is probable also that pathological changes occur in axis-cylinders and sheaths of the nerve-fibres themselves.

The present research has not yet investigated the changes in the pulp (if any) which may be associated with "zones of translucency" in the dentine, nor in cases in which the caries has undergone spontaneous arrest.

(B) *Conditions associated with Deep Caries.*

When, however, caries has advanced as far as the formation of a deep and extensive cavity in the dentine, then fresh structures are seen and must now be described.

The odontoblasts at the cervical region are enormously multiplied in point of numbers and layers. The cells themselves are not enlarged, but possess prominent oval nuclei which are much flattened laterally. Interposed here and there are small, hitherto undescribed, translucent globules, structureless and non-laminated, but similar in other respects to tiny calcospherite spherules. These are seen at the dentine border between the cells, and sometimes in Weil's layer. At the junction of the carious region with the primary or first-formed dentine the latest deposited dentine has, at its periphery, the globular appearances observed during developmental periods. It takes aniline dyes more deeply than the normal dentine, from which it is highly differentiated. This new tissue may be called "adventitious" dentine,—a term which includes several varieties to be hereafter noted. At the place where the carious tubules open into this freshly deposited layer, the odontoblasts are considerably shrunken, and pressed inward towards the pulp. They are disposed in one, or at most, two layers, and their peripheral poles (dentinal fibrils) are greatly enlarged and swollen. The layer of Weil is most marked here. Micrococci and bacilli infect the newly-formed tubes, and in some places expand them. And where tubular expansion has been effected, there the odontoblasts are absent, their places being occupied by a homogeneous mass of broken-down cells, with a few nuclei scattered about. Intense hyperæmia is distinguished by enlargement and tortuosity of the capillaries and arterioles, their engorgment with blood, and emigration of leucocytes.

As the thin sheet of adventitious dentine gradually widens out the odontoblasts become elongated, remaining all the time in one layer, their fibrils, each with its individual enveloping tube, being of normal size, and stretching across the new dentine at fairly regular intervals. The cells themselves are sometimes gathered into sheaves. With the widening of the sheet of dentine, they become smaller and shorter, and diminish rapidly in number, until they disappear altogether.

Meanwhile, the adventitious dentine presents the well-known appearance of areolation almost identical with that of the interglobular spaces. This areolar adventitious dentine is one of the most commonly recurring of varieties. The tubules which cross the spaces, sparingly filled with round and rod-shaped micro-organisms, enlarge greatly as they extend inward, and terminate with wide mouths at their pulpar extremities. This is probably caused by partial softening of the intertubular matrix. The structure of the pulp itself at this place is of the homogeneous character already noted. The dentine which fills the cornua of the pulp exhibits irregular formations, as if deposition had taken place in a hurried manner. Not only are nucleated cells with long processes embedded in the hard mass, but large lacunal spaces are frequent, each containing micrococci which have entered *via* the tubules of the primary dentine. In some instances this cellular dentine resembles somewhat the structure of sponge.

A bacteriological survey of the same specimens of hyperæmia and early stages of the lesions which Rothmann[1] has designated "partial acute pulpitis," and Wedl[2] "pulpitis acuta partialis," furnishes one with some valuable particulars as to the probable distribution of the micro-organisms in the pulp and surrounding tissue. Miller[3] has isolated, cultivated, and named the most important of the cocci and bacilli; here we have an opportunity of describing the probable routes of their invasion of the pulp itself. The micro-organisms, after their introduction into the pulp-cavity, are believed to make their way in chains, groups, or zooglia masses to the spaces between the odontoblast layer, the dentogenetic and ordinary pulp-cells on the one side, and the border of dentine on the other; and also to the interpolar (interfibrillar) spaces, and the intercellular intervals. Thence they travel apparently to the basal layer of Weil, although here they are not congregated in such large or such numerous masses. Whatever their point of entrance, they soon pass to some considerable distance along the line of junction of the hard and soft tissues.

Further, they are found in the substance of the pulp proper,

---

[1] Patho-Histologie der Zahnpulpa und Wurzelhart, 1889.

[2] Atlas zur Pathologie der Zahne, 1893, pp. 68, 69.

[3] Micro-organisms of the Human Mouth.

chiefly arranged along the walls of the blood-vessels, in their interiors (when empty), and in the perivascular tissues. Infection of the nerve fasciculi most probably does not take place. The micrococci predominate largely over the rod-shaped organisms. The central and peripheral portions of the adventitious dentine are crammed with micro-organisms, but when the odontoblast fibrils with their sheaths cross the areolations of this new deposit, no cocci can be found.

From these investigations, therefore, it will be seen that, as a result, one is unable to coincide with Arkövy's theory of the phagocytic function of the odontoblasts.[1] They certainly possess a granular appearance, but a search for any micro-organisms which have become incorporated in the substance of their protoplasm or nuclei is attended with negative results.

If the course of the disease is progressive, inflammatory foci appear. These consist of proliferated connective-tissue cells (macrophages), pulp-cells, and mono- or polynuclear leucocytes which have escaped from the numerous enlarged capillaries, all having been attracted together by a kind of positive chemotaxis. The foci are very pronounced, commence at first in one or both of the cornua of the pulp opposite the carious dentine, and, as a rule, ultimately suppurate and form localized abscesses. Rapid destruction of the pulp ensues, and the undermined dentine finally gives way in the majority of cases.

Sometimes a certain amount of fibrification of the cells lying in the immediate vicinity of the abscess occurs, and what might be termed a rudimentary abscess sac is developed. We are led to believe that this specialization and grouping of spindle-cells is not merely fortuitous, but a deliberate attempt on the part of the pulp to heal the lesion. The condition is observed in cases of chronic caries, the adventitious dentine being then deposited in layers, and presenting a characteristic fibrillar structure. On the border-line of the hard and soft parts, the connective-tissue structure of the dentine matrix is well brought out. Islands of dentine in the body of the pulp suggest that they are nothing more nor less than calcified bundles of connective-tissue fibres mixed with cells; the process of their formation being analogous to that of intramembranous ossification of bone.

Leaving aside the further study of these lesions, we rapidly pass to a brief consideration of the

---

[1] See Journal of British Dental Association, vol. xv., p. 602.

(C) *Conditions associated with Penetrating Caries.*

Limitation of time affords these notes opportunity of speaking of no more than two phases of one of the commonest conditions found in the mouth,—viz., idiopathic exposure of the pulp.

In median sections of teeth affected by acute caries, which has terminated in total inflammation and partial suppuration of the pulp, it is obvious that the cells appear degenerate altogether. Connective-tissue cells are broken down; the pulp-cells have become changed into indifferent cells with large square nuclei, and escaped leucocytes crowd the tissues. Even the odontoblasts themselves at the cervical region are metamorphosed into short cells with rounded nuclei, and at the coronal part are opaque, and seem to have undergone fatty or mucoid degeneration.

Finally, at the periphery of the pulp the small globular deposits, already mentioned, are found. The nerve-bundles have lost their definite structure, and though retained in position are evidently less translucent and more disorganized. There are no clear evidences of fatty degeneration in the sections under notice, although Wedl[1] describes this as existing in his sections of acute purulent pulpitis. The tissues are greatly condensed at the margins of the abscess cavity, the cells being short and fusiform, interlacing closely with each other. The blood-vessels are hyperæmic, and micrococci and bacilli are abundantly distributed throughout the tissue.

The last condition which we shall now consider is that of a phase of acute partial suppurative inflammation of the pulp, in which that organ has been subjected to the devitalizing action of arsenous acid for a period of not less than twelve hours.

In addition to appearances which denote the intensity of the inflammation,—hyperæmia, marked cellular infiltration, suppuration, and other changes common to acute inflammations in soft tissues,—a prominent feature is a large special form of dentinal deposit which is situated at the base of the carious opening into the pulp-chamber. It is irregularly rounded in shape. Its structure, in some places, is that of a more or less homogeneous matrix closely resembling that of hyaline cartilage; in other places it has a distinctly fibrous character. Distributed throughout, and with no attempt at uniformity of arrangement, are rounded or oval spaces containing one or more cells with large round nuclei. The cells vary very much in size, those near the pulp side being six or eight times

---

[1] Op. cit., p. 71.

as large as the smaller ones near the dentine side. Towards the pulp side rows of encapsuled cells exist, some being multinucleated. The surrounding pulp-tissue in immediate association with the new deposit consists chiefly of fusiform cells arranged in bundles interspersed with small round cells, the former possessing oval, and the latter round nuclei.

This particular form of cellular or hyaline adventitious dentine does not occur solely in acute inflammations of the pulp; it is also seen in chronic inflammation with hypertrophy (polypus) near the lower portion of the pulp-cavity. In this case it may be accompanied by new dentine, which has a pronounced laminar structure.

In conclusion, a preliminary study of the patho-histology of these lesions leads one to the following deductions:

(1) That nearly every degree of dentinal change is attended with hyperæmia, and cell-proliferation in the pulp-tissues, and, generally speaking, the formation of adventitious dentine.

(2) That the latter may have its origin as a conversion or secretion of the dentogenetic cells, producing, on the one hand, the areolar or laminar, or hyaline varieties, when the formative cells alone happen to be concerned; on the other, the fibrillar or cellular forms when odontoblasts or connective-tissue cells are by chance incorporated in the deposit.

(3) That there are no proofs that the cells called odontoblasts take any part in the production of the matrix of the new dentines, the term being therefore a misnomer; and

(4) That the new dentines, by a system of extension from the affected areas, may be just as much subjected to the peptonizing action of micro-organisms as the primary dentine of the tooth.—*Transactions of the Odontological Society of Great Britain.*

